# Association between vaginal microecological dysfunction and cervical lesion severity in postmenopausal women: a retrospective study

**DOI:** 10.3389/fmed.2026.1817031

**Published:** 2026-08-12

**Authors:** Fei Wang, Juan Wang, Wenxin Ha, Mengsha Wang, Yuqin Liu, Liehonga Wang

**Affiliations:** 1 Department of Gynaecology and Obstetrics, Qinghai Red Cross Hospital, Xining, Qinghai, China; 2 Department of Obstetrics and Gynecology, Mianyang Central Hospital, Mianyang, Sichuan, China; 3 Department of Gynaecology and Obstetrics, The Second People’s Hospital of Xining, Xining, Qinghai, China; 4 College of Clinical Medicine, Qinghai University, Xining, Qinghai, China

**Keywords:** cervical lesions, HPV, postmenopausal period, retrospective study, vaginal microecology

## Abstract

**Objective:**

To investigate the associations between vaginal microecological alterations and varying grades of cervical lesions in postmenopausal women, aiming to provide clinical evidence for the prevention and treatment of cervical lesions in this population.

**Methods:**

A total of 441 postmenopausal women who attended Qinghai Red Cross Hospital from January 2022 to January 2025 were enrolled. According to cervical histopathology, subjects were divided into a control group (*n* = 147, without cervical lesions) and a study group (*n* = 294). The study group was further categorized into low-grade squamous intraepithelial lesion (LSIL, *n* = 187), high-grade squamous intraepithelial lesion (HSIL, *n* = 72), and cervical cancer (CC, *n* = 35) subgroups. General demographic data were collected and analyzed. All participants underwent high-risk human papillomavirus (HR-HPV) testing, vaginal microecological functional assays—including pH, cleanliness, hydrogen peroxide (H_2_O_2_), leukocyte esterase (LE), sialidase (SNA), prolyl aminopeptidase (PIP), coagulase (GADP), β-glucuronidase (GUS), and N-acetyl-β-D-glucosaminidase (NAG)—and screening for common pathogens such as bacterial vaginosis (BV), vulvovaginal candidiasis (VVC), and Trichomonas vaginalis (TV). Intergroup differences were analyzed, and multivariate logistic regression was employed to identify independent independently associated with cervical lesions in postmenopausal women.

**Results:**

Baseline characteristics were comparable between groups (*P* > 0.05). The HR-HPV positive rate in the study group was significantly higher than in controls, increasing with lesion severity (*P* < 0.001). Among HR-HPV positive subjects, BV positivity was significantly higher than in HR-HPV negative subjects (*P* < 0.001), whereas VVC positivity was significantly lower than in HR-HPV negative counterparts (*P* = 0.002). HR-HPV positive women exhibited higher rates of abnormal vaginal pH ( > 4.5), decreased cleanliness (grades III–IV), and positivity for LE, GUS, and NAG compared to HR-HPV negative individuals (*P* < 0.001). Within the study groups, abnormal pH, decreased cleanliness, H_2_O_2_ positivity, LE, SNA, PIP, and BV detection rates were significantly elevated relative to controls, correlating positively with lesion severity (*P* < 0.001). Multivariate analysis identified HR-HPV infection (OR = 8.58), H_2_O_2_ positive (OR = 12.40), SNA (OR = 5.06), PIP (OR = 3.07), and BV infection (OR = 10.80) as independent independently associated with cervical lesions (*P* < 0.05).

**Conclusion:**

Cervical lesion development and progression in postmenopausal women are closely associated with persistent HR-HPV infection, vaginal microecological dysbiosis, and BV. H_2_O_2_ deficiency, BV infection, and HR-HPV positivity constitute the strongest risk factors. Monitoring and modulation of vaginal microecology may offer novel preventive and therapeutic strategies against cervical disease in this population.

## 1 Introduction

Cervical cancer ranks as the fourth most common malignancy among women worldwide, posing a significant health threat (1). Persistent infection with high-risk human papillomavirus (HR-HPV) is a necessary driver of cervical intraepithelial neoplasia and invasive cervical cancer (2). Although most HR-HPV infections clear spontaneously within 1–2 years through host immune responses, certain women, particularly postmenopausal women, are predisposed to persistent infection owing to immune senescence and hormonal alterations, ultimately progressing to cervical cancer (3). Identifying modifiable correlates influencing HR-HPV persistence and lesion progression is crucial for cervical cancer prevention. HPV is an epitheliotropic virus whose infection and replication intimately depend on the epithelial host microenvironment (4). Recently, increasing evidence has demonstrated that alterations in the female genital tract microbiota are closely associated with the development of gynecological malignancies (5). Beyond cervical cancer, dysbiosis of the reproductive tract microbiota has also been implicated in endometrial carcinogenesis through chronic inflammation, immune dysregulation, and altered metabolic signaling. A recent narrative review by Aquino et al. further emphasized the potential role of microbial homeostasis in gynecological cancers and highlighted the importance of microbiota-targeted preventive strategies (6). These findings support the concept that maintenance of a healthy vaginal microenvironment may represent a common protective mechanism against multiple gynecological malignancies. Accordingly, investigating vaginal microecological alterations in postmenopausal women may provide additional insights into cervical carcinogenesis. A healthy vaginal microbiome is dominated by H_2_O_2_-producing Lactobacillus species, sustaining an acidic environment that resists pathogen colonization via biotic antagonism, nutrient competition, and immunomodulation (7). Microecological imbalance is characterized by depletion of lactobacilli and overgrowth of anaerobic and facultative pathogens, reflected functionally by elevated vaginal pH, reduced cleanliness, and altered enzymatic activities including leukocyte esterase (LE), sialidase (SNA), and prolyl aminopeptidase (PIP) (8). This dysbiotic state may compromise cervical mucosal defenses, facilitating HR-HPV persistence and integration, thus accelerating cervical lesion development (9).

Postmenopausal women constitute a distinct demographic; ovarian failure induces precipitous estrogen decline, leading to vaginal epithelial atrophy, glycogen reduction, and diminished lactobacilli abundance (10). These changes increase susceptibility to microbial dysbiosis and pathogen colonization. Consequently, HR-HPV infection in this group is more prone to persistence and oncogenic transformation (11). With global aging, cervical cancer prevention in postmenopausal women demands heightened attention. Nevertheless, large-scale clinical research systematically elucidating the relationship between vaginal microecological function, common pathogen infections, and cervical lesion grades in postmenopausal women remains scarce. Therefore, this study aimed to compare HR-HPV status, vaginal microecological functional indicators, and pathogen prevalence between healthy postmenopausal women and patients with different cervical lesion grades. The goal is to delineate independently associated with inform clinical strategies for prevention and treatment of cervical lesions via modulation of vaginal microecological balance.

## 2 Materials and methods

### 2.1 Study population

**FIGURE 1:**
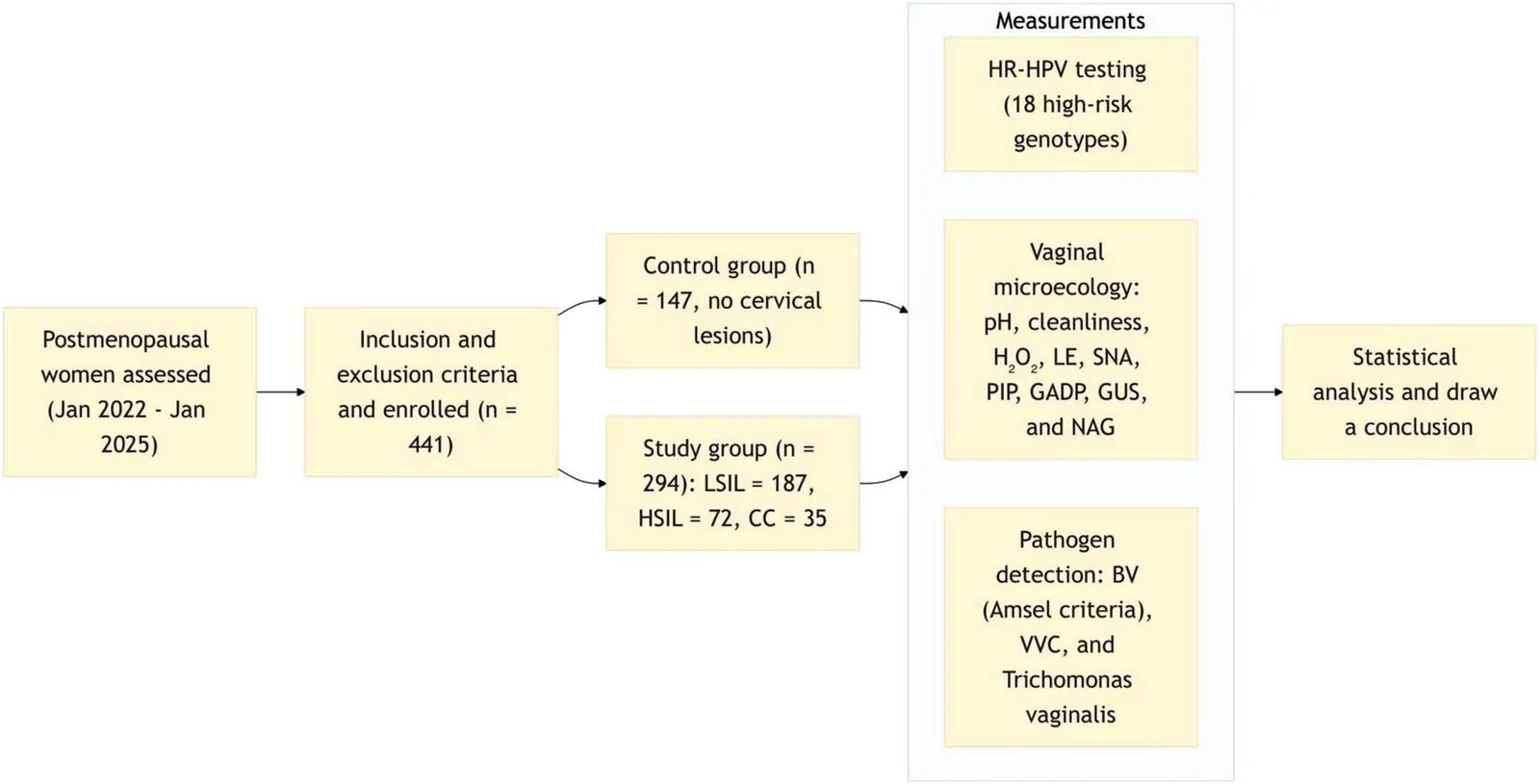
Flow diagram.

This was a retrospective cross-sectional study. This retrospective cross-sectional observational study was designed and reported in accordance with the Strengthening the Reporting of Observational Studies in Epidemiology (STROBE) Statement. Data were retrospectively collected from the medical records of postmenopausal women who attended the gynecology outpatient clinic and inpatient departments at Qinghai Red Cross Hospital from January 2022 to January 2025. All participants had undergone HR-HPV DNA testing (exposure), liquid-based cytology, and colposcopy-guided cervical biopsy (outcome) during the same clinical encounter; therefore, exposure and outcome were measured simultaneously. A total of 441 eligible women were included: 294 with histologically confirmed cervical lesions (study group), subdivided into LSIL (*n* = 187), HSIL (*n* = 72), and cervical cancer (CC, *n* = 35); and 147 controls without cervical lesions, whose data were extracted from the same source population during the same period. The selection process is detailed in Figure 1 (flow diagram). All patients had provided informed consent for the use of their data. The study was approved by the Ethics Committee of Qinghai Red Cross Hospital (KY-2024-43).

### 2.2 Inclusion and exclusion criteria

Inclusion criteria:(1) natural or surgical menopause for over one year; (2) study group patients with histologically confirmed LSIL, HSIL, or early-stage squamous cell cervical carcinoma; (3) no vaginal medication, douching, or sexual activity within 3 days prior to sample collection; (4) no treatment for cervical lesions, HPV infection, or vaginitis within 1 month before enrollment; (5) completion of a structured questionnaire on sexual behavior (lifetime number of sexual partners, age at first intercourse), socioeconomic status (education level, household income), contraceptive history, and history of prior sexually transmitted infections.

Exclusion criteria:

(1) pregnant or lactating women; (2) history of cervical conization or hysterectomy; (3) systemic or vaginal antibiotic or antiviral use within 4 weeks prior to enrollment; (4) systemic infectious diseases, autoimmune disorders, or malignancies at other sites; (5) incomplete clinical data; (6) current or recent (within 6 months) use of hormone replacement therapy; (7) diabetes mellitus with poor glycemic control or requiring insulin therapy; (8) known immunosuppressive conditions, including HIV infection, long-term corticosteroid use, or other immunosuppressive medications.

### 2.3 Methods

#### 2.3.1 Data collection

Demographic and clinical data including age, duration since menopause, gravida, parity, age at first intercourse, age at first delivery, smoking, and alcohol use were collected via questionnaires and medical records review.

#### 2.3.2 Vaginal microecological assessment

During gynecological examination, sterile swabs were used to obtain vaginal secretions from the lateral vaginal wall (lower third). Vaginal microecological assessment is routinely performed in our department for postmenopausal women undergoing cervical lesion evaluation or HR-HPV screening, according to the standard clinical protocol of Qinghai Red Cross Hospital. Therefore, all laboratory data included in this retrospective analysis were obtained from routine clinical examinations rather than additional research-specific procedures.

(1) Morphological evaluation: Samples were smeared onto slides, air-dried, Gram-stained, and examined under oil immersion microscopy by two experienced pathologists who were blinded to the participants’ clinical group allocation. Vaginal flora, cleanliness (grades I–II as normal, III–IV as abnormal), clue cells, Trichomonas, and fungal elements were assessed. To ensure consistency, inter-observer agreement was periodically evaluated, and any discrepancies were resolved by consensus. (2) Functional assays: Using a vaginal infection combined test kit (dry chemistry enzymatic method; Jiangsu Bioperfectus Technologies Co., Ltd., Taizhou, China; Cat. No. SS10101; NMPA registration No. Su Xie Zhu Zhun 20152401398), vaginal pH ( > 4.5 considered abnormal), hydrogen peroxide (H_2_O_2_, positive indicating impaired lactobacilli function and imbalanced microbiota, which may increase infection risk), leukocyte esterase (LE, an inflammatory marker), sialidase (SNA, a bacterial vaginosis-associated enzyme), β-glucuronidase (GUS, a marker of aerobic bacterial infection), and N-acetyl-β-D-glucosaminidase (NAG, an epithelial damage indicator) were measured following the manufacturer’s protocol. Sensitivity and specificity data for these assays were not provided by the manufacturer. All functional assays were performed by laboratory technicians who were blinded to the clinical group allocation. Internal quality control samples were included in each analytical run to verify assay performance.

(3) Pathogen diagnosis: BV was diagnosed per Amsel criteria requiring three out of four signs (12): presence of clue cells, pH > 4.5, positive amine test, and thin homogeneous discharge. VVC diagnosis was based on microscopy detecting pseudohyphae or spores. TV infection was confirmed by visualization of motile trichomonads. These microscopic assessments were also performed by the blinded pathologists.

#### 2.3.3 HR-HPV detection

Cervical exfoliated cells were collected with a cervical brush inserted into the endocervical canal, rotated five times clockwise, and preserved in liquid medium. Polymerase chain reaction (PCR) amplification combined with reverse dot blot hybridization identified 18 HR-HPV genotypes (16, 18, 31, 33, 35, 39, 45, 51, 52, 53, 56, 58, 59, 66, 68, 73, 82, 83). Detection of any subtype constituted a positive result (13).

#### 2.3.4 Diagnosis of cervical lesions

All subjects underwent liquid-based cytology examination. Those who were HR-HPV positive, had abnormal cytology, or clinical suspicion underwent colposcopy. Under colposcopy guidance, suspicious areas were biopsied, and cervical canal curettage was performed if necessary. Pathological diagnosis was independently reviewed by two senior pathologists and made according to the WHO Classification of Female Genital Tumors, categorized as: normal or inflammation, LSIL (including CIN I), HSIL (including CIN II and CIN III), and invasive cervical cancer (14). If multiple biopsy sites were inconsistent, the highest grade lesion was taken as the final diagnosis.

### 2.4 Statistical analysis

Data were analyzed using SPSS 29.0 (IBM Corp., Armonk, NY, USA). Normality of continuous variables was assessed using the Shapiro-Wilk test. Variables conforming to a normal distribution were expressed as mean ± standard deviation (SD) and compared using one-way analysis of variance (ANOVA) with *post-hoc* pairwise comparisons adjusted by Bonferroni correction, or Student’s *t*-test for two-group comparisons. Categorical variables were expressed as frequencies (percentages) and compared using the chi-square test or Fisher’s exact test; for pairwise comparisons of categorical data among multiple groups, Bonferroni-adjusted *p-*values were applied. Variables with a *p*-value < 0.10 in univariate analysis were considered for inclusion in a multivariate logistic regression model to identify independent independently associated with cervical lesions. Before modeling, multicollinearity among candidate variables was assessed using variance inflation factors (VIFs), with VIF > 10 considered indicative of significant collinearity. Variable selection was performed using a forward stepwise method based on the likelihood ratio test, with entry and removal criteria of *p* < 0.05 and *p* > 0.10, respectively. Model goodness-of-fit was evaluated using the Hosmer-Lemeshow test, and discrimination was assessed by the area under the receiver operating characteristic curve (AUC). Potential interactions between HR-HPV infection status and other covariates were examined by including interaction terms in the model; no significant interactions were detected. Missing data were minimal ( < 5% for any variable) and were handled by complete case analysis. Given the small sample size of the cervical cancer subgroup (*n* = 35), subgroup-specific regression analyses were not performed, and odds ratios from the overall model should be interpreted with caution. A two-tailed *p*-value < 0.05 was considered statistically significant.

## 3 Results

### 3.1 Comparison of general data

A total of 441 postmenopausal women were enrolled, including 147 in the control group and 294 in the study group (187 LSIL, 72 HSIL, 35 cervical cancer). No significant differences were found between the two groups in age, years since menopause, gravidity, parity, age at first sexual intercourse, age at first birth, smoking history, or drinking history (*P* > 0.05), indicating balanced baseline data with comparability. Mean age was (53.3 ± 5.8) years in controls and (54.8 ± 6.1) years in study group; years since menopause were (5.2 ± 4.1) and (5.5 ± 4.3) respectively, with no significant difference (*P* > 0.05). Regarding gravidity ≥ 3, controls were 38.1% (56/147), study group 38.4% (113/294) (*P* = 0.885); parity ≥ 3 was 22.4% (33/147) vs. 20.1% (59/294) (*P* = 0.948). Age at first sexual intercourse < 18 years: 36.1% (controls) vs. 38.1% (study), no difference (χ^2^ = 0.288, *P* = 0.591); age at first birth ≤ 22 years: 22.4% vs. 20.1% (*P* = 0.754). Smoking rates (12.9% vs. 13.6%) and drinking rates (10.2% vs. 9.9%) showed no significant difference (*P* > 0.05) (see Table 1).

**TABLE 1:** Comparison of baseline data between two groups.

Clinical data	Control group (*n* = 147)	Study group (*n* = 294)	*t/X_2_*	*P*
Years since menopause (years)	5.2 ± 4.1	5.5 ± 4.3	−0.832	0.406
Age (years)	53.3 ± 5.8	54.8 ± 6.1	−0.714	0.476
Gravidity (times)				
< 3	91 (61.9)	181 (61.6)	0.021	0.885
≥ 3	56 (38.1)	113 (38.4)		
Parity (times)				
<3	114 (77.6)	235 (79.9)	0.004	0.948
≥ 3	33 (22.4)	59 (20.1)		
Age at first sexual intercourse (years)				
< 18	53 (36.1)	112 (38.1)	0.288	0.591
≥ 18	94 (63.9)	182 (61.9)		
Age at first birth (years)				
≤ 22	33 (22.4)	59 (20.1)	0.098	0.754
> 22	114 (77.6)	235 (79.9)		
Smoking history				
No	128 (87.1)	254 (86.4)	0.102	0.749
Yes	19 (12.9)	40 (13.6)		
Drinking history				
No	132 (89.8)	265 (90.1)	0.011	0.917
Yes	15 (10.2)	29 (9.9)		

### 3.2 Comparison of HR-HPV positivity rates between groups

The HR-HPV positivity rate was 20.4% (30/147) in controls and 77.2% (227/294) in the study group, with a significant difference (χ^2^ = 160.458, *P* < 0.001). Subgroup rates within the study group were: LSIL 70.1% (131/187), HSIL 87.5% (63/72), and cervical cancer 94.3% (33/35), showing a progressive increase with lesion severity (see Table 2).

**TABLE 2:** Comparison of HR-HPV positivity rates between groups.

Group	n	HR-HPV positivity rate	HR-HPV negative rate	*X* ^2^	*P*
Control group	147	30 (20.4)	117 (79.6)	160.458	< 0.001
Study group	294	227 (77.2)	67 (22.8)		

### 3.3 Relationship between vaginal microecological function and HR-HPV infection

Significant differences in vaginal microecological indicators were observed between HR-HPV positive and negative groups. The HR-HPV positive group had pH > 4.5 in 68.1% of cases, significantly higher than the 35.3% in the negative group (*P* < 0.001). Cleanliness grades III–IV were 76.3% vs. 40.2% (*P* < 0.001). The LE positive rate was 80.2% vs. 57.1% (*P* < 0.001). GUS positivity was 62.3% vs. 39.1% (*P* < 0.001). NAG positivity was 65.4% vs. 28.3% (*P* < 0.001). No significant difference was found for H_2_O_2_, SNA, GADP, or PIP positivity between groups (*P* > 0.05) (see Table 3).

**TABLE 3:** Relationship between vaginal microecological function and HR-HPV infection.

Index	HR-HPV infection		*X* ^2^	*P*
	Positives (*n* = 257)	Negative (*n* = 184)		
PH				
3.8–4.5	82 (31.9)	119 (64.7)	49.213	< 0.001
> 4.5	175 (68.1)	65 (35.3)		
Cleanliness grades				
I–II	61 (23.7)	110 (59.8)	77.342	< 0.001
III–IV	196 (76.3)	74 (40.2)		
H2O2				
−	102 (39.7)	75 (40.8)	0.108	0.743
+	155 (60.3)	109 (59.2)		
LE				
−	51 (19.8)	79 (42.9)	32.147	< 0.001
+	206 (80.2)	105 (57.1)		
SNA				
−	118 (45.9)	93 (50.5)	0.876	0.349
+	139 (54.1)	91 (49.5)		
GADP				
−	133 (51.8)	99 (53.8)	0.294	0.588
+	124 (48.2)	85 (46.2)		
PIP				
−	123 (47.9)	88 (47.8)	0.034	0.854
+	134 (52.1)	96 (52.2)		
GUS				
−	97 (37.7)	112 (60.9)	19.506	< 0.001
+	160 (62.3)	72 (39.1)		
NAG				
−	89 (34.6)	132 (71.7)	84.625	< 0.001
+	168 (65.4)	52 (28.3)		

### 3.4 Relationship between vaginal pathogens and HR-HPV infection

The BV positive rate was 70.0% in the HR-HPV positive group, significantly higher than the 29.9% in the negative group (*P* < 0.001). The VVC positive rate was lower in the HR-HPV positive group (24.9% vs. 45.1%, *P* = 0.002). TV positive rates showed no significant difference (40.1% vs. 39.7%, *P* = 0.700) (see Table 4).

**TABLE 4:** Relationship between vaginal pathogens and HR-HPV infection.

Index	HR-HPV Infection		*X* ^2^	*P*
	Positives (*n* = 484)	Negative (*n* = 251)		
BV				
−	77 (30.0)	129 (70.1)	86.338	< 0.001
+	180 (70.0)	55 (29.9)		
VVC				
−	193 (75.1)	101 (54.9)	9.874	0.002
+	64 (24.9)	83 (45.1)		
TV				
−	154 (59.9)	111 (60.3)	0.148	0.700
+	103 (40.1)	73 (39.7)		

### 3.5 Comparison of vaginal microecological function between study and control groups

Significant differences in multiple vaginal microecological indices were noted between groups. pH > 4.5 occurred in 11.6% of the control group, and 50.8%, 79.2%, and 85.7% in the LSIL, HSIL, and CC subgroups respectively, increasing with lesion severity (*P* < 0.001). Cleanliness III–IV grades were 54.4% in controls vs. 57.2%, 70.8%, and 88.6% in the LSIL, HSIL, and CC groups (*P* < 0.001). H_2_O_2_ positivity was 53.7% among controls vs. 40.1%, 65.3%, and 82.9% in the respective subgroups (*P* < 0.001). LE positivity was 44.2% vs. 62.6%, 83.3%, and 88.6% (*P* < 0.001). SNA positivity was 17.0% in controls vs. 49.7%, 66.7%, and 65.7% in the study group (*P* < 0.001). PIP positivity was 20.4% in controls vs. 50.3%, 59.7%, and 62.9% in the study group (*P* < 0.001). No significant differences were noted for GADP, GUS, and NAG positivity between groups (*P* > 0.05) (see Table 5).

**TABLE 5:** Comparison of vaginal microecological function between study and control groups.

Index	*N*	Control group (*n* = 147)	Study group (*n* = 294)			*X* ^2^	*P*
			LSIL (*n* = 187)	LSIL (*n* = 72)	CC (*n* = 35)		
PH							
3.8–4.5	242	130 (88.4)	92 (49.2)	15 (20.8)	5 (14.3)	126.234	< 0.001
> 4.5	199	17 (11.6)	95 (50.8)	57 (79.2)	30 (85.7)		
Cleanliness grades							
I–II	172	67 (45.6)	80 (42.8)	21 (29.2)	4 (11.4)	18.012	< 0.001
III–IV	269	80 (54.4)	107 (57.2)	51 (70.8)	31 (88.6)		
H2O2							
−	211	68 (46.3)	112 (59.9)	25 (34.7)	6 (17.1)	29.175	< 0.001
+	230	79 (53.7)	75 (40.1)	47 (65.3)	29 (82.9)		
LE							
−	168	82 (55.8)	70 (37.4)	12 (16.7)	4 (11.4)	44.108	< 0.001
+	273	65 (44.2)	117 (62.6)	60 (83.3)	31 (88.6)		
SNA							
−	252	122 (83.0)	94 (50.3)	24 (33.3)	12 (34.3)	67.851	< 0.001
+	189	25 (17.0)	93 (49.7)	48 (66.7)	23 (65.7)		
GADP							
−	255	97 (66.0)	111 (59.4)	32 (44.4)	15 (42.9)	3.648	0.143
+	186	50 (34.0)	76 (40.6)	40 (55.6)	20 (57.1)		
PIP							
−	252	117 (62.6)	93 (46.0)	29 (40.3)	13 (37.1)	49.023	< 0.001
+	186	30 (20.4)	94 (50.3)	43 (59.7)	22 (62.9)		
GUS							
−	217	92 (62.6)	86 (46.0)	27 (37.5)	12 (34.3)	6.029	0.110
+	224	55 (37.4)	101 (54.0)	45 (62.5)	23 (65.7)		
NAG							
−	217	83 (56.5)	93 (49.7)	30 (41.7)	11 (31.4)	3.865	0.276
+	224	64 (43.5)	94 (50.3)	42 (58.3)	24 (68.6)		

### 3.6 Pathogen detection comparison between groups

Significant differences in BV detection rates were observed. BV positivity was 10.2% in controls vs. 52.9%, 62.5%, and 80.0% in the LSIL, HSIL, and CC groups respectively, increasing with lesion progression (*P* < 0.001). VVC detection showed no significant difference among groups (29.9% controls vs. ∼30% study, *P* > 0.05). TV detection rates were also not significantly different (30.6% controls vs. 32.6–51.4% study, *P* > 0.05) (see Table 6).

**TABLE 6:** Pathogen detection comparison between groups.

Index	Control group (n = 147)	Study group (*n* = 294)			*X* ^2^	*P*
		LSIL (*n* = 187)	HSIL (*n* = 72)	CC (*n* = 35)		
BV	15 (10.2)	99 (52.9)	45 (62.5)	28 (80.0)	103.073	< 0.001
VVC	44 (29.9)	62 (33.2)	27 (37.5)	10 (28.6)	0.672	0.672
TV	45 (30.6)	61 (32.6)	29 (40.3)	18 (51.4)	6.752	0.080

### 3.7 Logistic multivariate regression analysis of cervical lesion risk factors

Multivariate logistic regression results indicated independent independently associated with cervical lesions in postmenopausal women: HR-HPV infection (OR = 8.584, 95% CI: 2.639–27.925), H_2_O_2_ positivity (OR = 12.398, 95% CI: 3.713–41.412), SNA positivity (OR = 5.055, 95% CI: 1.584–16.130), PIP positivity (OR = 3.066, 95% CI: 1.178–7.982), and BV infection (OR = 10.802, 95% CI: 2.592–45.003) (*P* < 0.05). Among these, H2O2 positivity carried the highest risk, followed by BV infection and HR-HPV infection. LE positivity, pH > 4.5, and abnormal cleanliness had no statistical significance in this study (*P* > 0.05) (see Table 7).

**TABLE 7:** Logistic multivariate regression analysis of cervical lesion risk factors.

Index	B	SE	Wald	*P*	OR (95% CI)
HR-HPV	2.150	0.602	12.752	0.001	8.584 (2.639–27.925)
LE	0.580	0.615	0.889	0.346	1.786 (0.535–5.962)
pH	−0.320	0.548	0.341	0.559	0.726 (0.248–2.124)
H_2_O_2_	4.820	0.935	26.586	< 0.001	12.398 (3.713–41.412)
Cleanliness grades	−0.850	0.745	1.302	0.254	0.427 (0.099–1.842)
SNA	1.620	0.592	7.486	0.006	5.055 (1.584–16.130)
PIP	1.120	0.488	5.267	0.022	3.066 (1.178–7.982)
BV	2.380	0.728	10.691	0.001	10.802 (2.592–45.003)

## 4 Discussion

This cross-sectional study involving 441 postmenopausal women systematically investigated the associations among HR-HPV infection, vaginal microecological functional markers, common pathogens, and the severity of cervical lesions. Our findings demonstrate a significant increasing trend in HR-HPV positivity rate, vaginal microecological disturbance indicators, and bacterial vaginosis (BV) detection rate as cervical lesions progress from LSIL to HSIL and invasive cervical cancer (CC). However, due to the cross-sectional design, causality cannot be inferred. Multivariate analysis confirmed HR-HPV infection, H_2_O_2_ positive, SNA, PIP, and BV infection as independent independently associated with cervical lesions in postmenopausal women, with H_2_O_2_ positivity exhibiting the strongest association (OR = 12.398), followed by BV infection (OR = 10.802) and HR-HPV infection (OR = 8.584). These results offer new clinical evidence to elucidate the pathogenesis of cervical lesions in this population.

Consistent with the global consensus that persistent HR-HPV infection is a necessary cause for cervical carcinogenesis, the HR-HPV positivity rate increased markedly from 20.4% in controls to 70.1% in LSIL, 87.5% in HSIL, and 94.3% in CC groups. Postmenopausal women exhibit a diminished ability to clear HR-HPV due to immunosenescence and hormonal changes, making persistent infection and progression more likely (15). This aligns with previous studies indicating two peaks of HPV infection risk during a woman’s lifespan: reproductive age and perimenopause, linked to declining estrogen levels and impaired vaginal mucosal defenses (16, 17).

Evaluation of vaginal microecological function revealed significant correlations between multiple indicators and cervical lesion severity. Notably, H_2_O_2_ positivity, mainly produced by Lactobacillus species, showed a paradoxical pattern: it decreased in LSIL but increased substantially in HSIL and CC groups. This may reflect an early compensatory response failure of Lactobacillus as cervical lesions advance and vaginal dysbiosis worsens, consistent with Liu et al. who reported a negative correlation between total Lactobacillus 16S rRNA expression and postmenopausal cervical lesion progression (18). Similarly, Korean studies have documented reduced Lactobacillus abundance and increased Gardnerella and Atopobium species in HPV-infected postmenopausal women compared to premenopausal counterparts (19). These findings underscore diminished Lactobacillus dominance and increased microbial diversity as key microecological bases for persistent HPV infection and lesion progression post-menopause.

SNA and PIP, enzymatic markers of BV-associated anaerobes such as Gardnerella, Prevotella, and Mobiluncus, also increased significantly with lesion severity. Their positive rates rose from ∼17–20% in controls to over 60% in HSIL and CC groups, and both showed independent risk associations (OR = 5.055 for SNA; OR = 3.066 for PIP). This accords with Luo et al.’s large cohort study linking SNA positivity and HR-HPV infection with cytological abnormalities in menopausal women (20). These enzymes degrade cervical mucus and disrupt mucosal barriers, promoting localized inflammation and facilitating HPV persistence via activation of NF-κB and related pathways (21).

Although LE positivity, elevated vaginal pH ( > 4.5), and abnormal cleanliness were higher in HR-HPV-positive and cervical lesion groups, they did not retain significance in multivariate models, suggesting their effects may be mediated or overshadowed by stronger factors such as H_2_O_2_, BV, and HR-HPV infection. These parameters likely reflect non-specific vaginal inflammation linked to microbial imbalance rather than direct pathogenic contributors.

Importantly, BV detection exhibited a marked increase along disease progression—from 10.2% in controls to 80.0% in CC—with independent risk significance (OR = 10.802). BV, characterized by depletion of lactobacilli and overgrowth of anaerobes, produces metabolic enzymes (e.g., sialidase, aminopeptidase) that compromise cervical mucus and elicit inflammatory responses, synergistically enhancing HR-HPV persistence and integration, thereby accelerating carcinogenesis (22). Our data further confirm the strong coexistence of BV and HR-HPV infection (70.0% BV positivity in HR-HPV-positive versus 29.9% in negative cases, *P* < 0.001). Conversely, VVC and TV infection rates did not differ significantly across groups, suggesting that anaerobic bacterial dysbiosis rather than fungal or protozoal infections plays a dominant role in cervical lesion progression post-menopause.

This study focused on postmenopausal women, a clinically significant group characterized by estrogen-deficiency-induced vaginal atrophy, glycogen depletion, reduced lactobacilli, and elevated pH, which together contribute to a fragile vaginal microecological balance (23). These microecological changes have been associated with an increased likelihood of persistent HR-HPV infection and a higher prevalence of cervical lesions, although the cross-sectional design of this study precludes any inference of causality. In our analysis, HR-HPV infection, H_2_O_2_ positivity, and BV infection each showed odds ratios exceeding 8, indicating strong associations with the presence of cervical lesions in this population. However, these findings reflect associations rather than direct causal relationships. Given that targeted antiviral therapies for HR-HPV remain limited, microecological modulation through lactobacilli supplementation and treatment of BV may represent a potential strategy for restoring vaginal homeostasis; yet, whether such interventions can reduce cervical lesion risk requires confirmation in prospective studies (24, 25). Therefore, our research results indicate that vaginal microecological imbalance is associated with HR-HPV infection and cervical lesions to some extent.

Restoration of vaginal Lactobacillus dominance through probiotic supplementation has emerged as a promising strategy for improving vaginal microecological balance. Previous clinical studies have suggested that probiotic administration may reduce bacterial vaginosis recurrence, improve vaginal acidity, and potentially facilitate HR-HPV clearance by enhancing local immune responses. Although current evidence remains insufficient to support probiotics as a standard therapy for preventing cervical lesions, our findings provide additional support for future prospective studies evaluating microbiota-targeted interventions in postmenopausal women. In addition, increasing evidence suggests that the estrobolome—the collection of gut microbial genes involved in estrogen metabolism—may indirectly influence the vaginal microbiome through regulation of systemic estrogen availability. Alterations in estrobolome activity after menopause may further reduce estrogen bioavailability, thereby contributing to vaginal epithelial atrophy, depletion of Lactobacillus species, elevated vaginal pH, and increased susceptibility to persistent HR-HPV infection. Although the present study did not evaluate gut microbiota or estrobolome composition, this emerging host–microbiome interaction deserves further investigation in future longitudinal studies.

Compared with previous studies, our research provides several novel contributions. First, we specifically focused on postmenopausal women, a population that remains underrepresented in studies of vaginal microecology and cervical lesions. Second, instead of evaluating only HR-HPV infection or bacterial vaginosis, we comprehensively assessed functional vaginal microecological indicators, including enzymatic biomarkers and vaginal cleanliness, together with common vaginal pathogens. Third, we identified H2O2 deficiency, bacterial vaginosis, and HR-HPV infection as the strongest independent factors associated with cervical lesions, thereby providing a more comprehensive understanding of the relationship between vaginal microecological dysfunction and cervical disease progression in postmenopausal women.

### 4.1 Limitations

This study has several limitations: (1) its cross-sectional design precludes causal or temporal inference between vaginal microecological alterations and cervical lesion development; (2) participants were recruited from a single center which may limit generalizability due to selection bias; (3) microecological assessments relied on functional enzymatic and conventional pathogen detection rather than molecular methods (e.g., 16S rRNA sequencing), restricting insights into detailed microbiome composition and diversity; (4) absence of longitudinal follow-up limited evaluation of dynamic changes in vaginal flora and HR-HPV infection status; (5) lack of HPV genotyping data for all subjects and inability to assess HPV persistence; (6) no adjustment for potential confounders such as hormone therapy, diabetes, and HPV vaccination status. And residual confounding cannot be ruled out.

## 5 Conclusion and future directions

In conclusion, this study highlighted the close correlation of HR-HPV infection, vaginal microecological dysfunction, and BV infection with cervical lesion occurrence and progression in postmenopausal women. Notably, the risk effects of Lactobacillus functional decline and BV infection surpass those of HR-HPV alone, underscoring the independent and critical role of vaginal microecological imbalance in cervical carcinogenesis after menopause. Clinically, maintaining Lactobacillus dominance and correcting BV represent potential targets for future preventive strategies, but these hypotheses require validation in prospective cohort studies and randomized controlled trials. Prospective cohort studies are warranted to delineate the temporal relationships among microecological changes, persistent HR-HPV infection, and cervical lesion progression, while randomized controlled trials should evaluate the efficacy of microecological modulators in preventing and managing cervical lesions in this high-risk group.

## Data Availability

The original contributions presented in this study are included in this article/supplementary material, further inquiries can be directed to the corresponding authors.

## References

[1] Sung H Ferlay J Siegel RL Laversanne M Soerjomataram I Jemal A et al. Global cancer statistics 2020: globocan estimates of incidence and mortality worldwide for 36 cancers in 185 countries. *CA Cancer J Clin.* (2021) 71:209–49. 10.3322/caac.21660 33538338

[2] Walboomers JM Jacobs MV Manos MM Bosch FX Kummer JA Shah KV et al. Human papillomavirus is a necessary cause of invasive cervical cancer worldwide. *J Pathol* (1999) 189:12–9. 10.1002/(SICI)1096-9896(199909)189:1<12::AID-PATH431>3.0.CO;2-F 10451482

[3] Castle PE Schiffman M Herrero R Hildesheim A Rodriguez AC Bratti MC et al. A prospective study of age trends in cervical human papillomavirus acquisition and persistence in Guanacaste, Costa Rica. *J Infect Dis.* (2005) 191:1808–16. 10.1086/428779 15871112

[4] Alizhan D Ukybassova T Bapayeva G Aimagambetova G Kongrtay K Kamzayeva N et al. Cervicovaginal microbiome: Physiology, age-related changes, and protective role against human papillomavirus infection. *J Clin Med.* (2025) 14:1521. 10.3390/jcm14051521 40094958 PMC11900180

[5] Brotman RM. Vaginal microbiome and sexually transmitted infections: an epidemiologic perspective. *J Clin Invest*. (2011) 121:4610–7. 10.1172/JCI57172 22133886 PMC3225992

[6] Aquino CI La Vecchia M Pasolli E Sala G Ligori A Boldorini R et al. Decoding the microbial landscape of endometrial cancer: a case-control study. *BMC Microbiol.* (2026) 26:492. 10.1186/s12866-026-05017-4 41975253 PMC13200458

[7] O’Hanlon DE Moench TR Cone RA. Vaginal pH and microbicidal lactic acid when lactobacilli dominate the microbiota. *PLoS One*. (2013) 8:e80074. 10.1371/journal.pone.0080074 24223212 PMC3819307

[8] Li Y Wu X. Vaginal microbiome distinction in women with HPV+, cervical intraepithelial neoplasia, and cervical cancer, a retrospective study. *Front Cell Infect Microbiol.* (2025) 14:1483544. 10.3389/fcimb.2024.1483544 39897478 PMC11782028

[9] Mitra A MacIntyre DA Lee YS Smith A Marchesi JR Lehne B et al. Cervical intraepithelial neoplasia disease progression is associated with increased vaginal microbiome diversity. *Sci Rep*. (2015) 5:16865. 10.1038/srep16865 26574055 PMC4648063

[10] Alvisi S Ceccarani C Foschi C Baldassarre M Lami A Severgnini M et al. Effect of ospemifene on vaginal microbiome in postmenopausal women with vulvovaginal atrophy. *Menopause.* (2023) 30:361–9. 10.1097/GME.0000000000002150 36727789

[11] Tachedjian G Aldunate M Bradshaw CS Cone RA. The role of lactic acid production by probiotic Lactobacillus species in vaginal health. *Res Microbiol* (2017) 168:782–92. 10.1016/j.resmic.2017.04.001 28435139

[12] Amsel R Totten PA Spiegel CA Chen KC Eschenbach D Holmes KK. Nonspecific vaginitis. Diagnostic criteria and microbial and epidemiologic associations. *Am J Med*. (1983) 74:14–22. 10.1016/0002-9343(83)91112-9 6600371

[13] Sun P Song Y Ruan G Mao X Kang Y Dong B et al. Clinical validation of the PCR-reverse dot blot human papillomavirus genotyping test in cervical lesions from Chinese women in the Fujian province: a hospital-based population study. *J Gynecol Oncol*. (2017) 28:e50. 10.3802/jgo.2017.28.e50 28657218 PMC5540716

[14] Hodge JC Netto GJ Rekhi B Cooper WA Eden M Field AS et al. WHO Classification of Tumours: evolution of a global resource in the molecular era. *Lancet Oncol*. (2025) 26:410–3. 10.1016/S1470-2045(24)00709-5 40179904

[15] Gravitt PE. Evidence and impact of human papillomavirus latency. *Open Virol J*. (2012) 6:198–203. 10.2174/1874357901206010198 23341855 PMC3547385

[16] Avsaroglu E Kaleli B Kilic D Kaleli I Guler TA. Decrease in lactobacilli in the vaginal microbiota is independently associated with HPV Persistence in women with high-risk HPV infection. *Cureus.* (2023) 15:e50907. 10.7759/cureus.50907 38259378 PMC10801280

[17] Brusselaers N Shrestha S de Wijgert J Verstraelen H. Vaginal dysbiosis and the risk of human papillomavirus and cervical cancer: systematic review and meta-analysis. *Am J Obstet Gynecol* (2019) 221:9e–18e. 10.1016/j.ajog.2018.12.011 30550767

[18] Liu J Song J Yang Q Wang Y. Correlation between *Lactobacillus* and expression of E-cadherin, β-catenin, N- cadherin, and Vimentin in postmenopausal cervical lesions. *Ann Palliat Med.* (2022) 11:135–45. 10.21037/apm-21-3581 35144405

[19] Wang Y Chen H Chen Y Lu Y Wei B Wu Z et al. Age-specific vaginal microecological dysbiosis associated with HPV infection: A large -scale cross-sectional study with targeted functional sequencing validation. *Front Cell Infect Microbiol.* (2026) 15:1722367. 10.3389/fcimb.2025.1722367 41647262 PMC12868268

[20] Mac Eochagain C Power R Parker I Brennan D. HPV vaccination among seropositive, DNA negative cohorts: A systematic review & meta-analysis. *J Gynecol Oncol.* (2022) 33:e24. 10.3802/jgo.2022.33.e24 35128855 PMC9024181

[21] Yang J Gao M Wang D Li Z Li S Zhou M et al. The role of enterococcus faecium in the synergistic clearance of high-risk HPV: A clinical study on cervicovaginal microbiota regulation. *Cancer Med.* (2026) 15:e72063. 10.1002/cam4.72063 42337942 PMC13290700

[22] Gillet E Meys JF Verstraelen H Bosire C De Sutter P Temmerman M et al. Bacterial vaginosis is associated with uterine cervical human papillomavirus infection: a meta-analysis. *BMC Infect Dis*. (2011) 11:10. 10.1186/1471-2334-11-10 21223574 PMC3023697

[23] Tsuboi I Inoue S Hirayama T Mitsui Y Watanabe M Hirakawa H et al. Gut, vaginal, and urinary microbiome alterations in women with genitourinary syndrome of menopause: A systematic review. *Maturitas.* (2026) 211:109031. 10.1016/j.maturitas.2026.109031 42341424

[24] Ji L Wu J Zhou Y Pu X Wang X Xu B et al. A prospective cohort study on the association between cervical microenvironmental factors and the efficacy of treating high-risk human papillomavirus infection comorbid with cervical diseases. *Front Cell Infect Microbiol.* (2026) 16:1734088. 10.3389/fcimb.2026.1734088 41884530 PMC13008986

[25] Nazarova V Kamzayeva N Kozhakhmetov S Kushugulova A Terzic M Bapayeva G et al. Tiered functional screening identifies an autochthonous vaginal *Lactiplanti bacillus* plantarum strain with probiotic potential. *Microorganisms.* (2026) 14:1526. 10.3390/microorganisms14071526 42514031 PMC13414338

